# Hydrogen Sulfide Gas Exposure Induces Necroptosis and Promotes Inflammation through the MAPK/NF-*κ*B Pathway in Broiler Spleen

**DOI:** 10.1155/2019/8061823

**Published:** 2019-07-31

**Authors:** Qianru Chi, Dongxu Wang, Xueyuan Hu, Shiping Li, Shu Li

**Affiliations:** ^1^College of Veterinary Medicine, Northeast Agricultural University, Harbin 150030, China; ^2^College of Animal Science and Technology, Northeast Agricultural University, Harbin 150030, China

## Abstract

Hydrogen sulfide (H_2_S) is one of the main pollutants in the atmosphere, which is a serious threat to human health. The decomposition of sulfur-containing organics in chicken houses could produce a large amount of H_2_S, thereby damaging poultry health. In this study, one-day-old broilers were selected and exposed to 4 or 20 ppm of H_2_S gas (0-3 weeks: 4 ± 0.5 ppm, 4-6 weeks: 20 ± 0.5 ppm). The spleen samples were collected immediately after the chickens were euthanized at 2, 4, and 6 weeks. The histopathological and ultrastructural observations showed obvious necrosis characteristics of H_2_S-exposed spleens. H_2_S exposure suppressed GSH, CAT, T-AOC, and SOD activities; increased NO, H_2_O_2_, and MDA content and iNOS activity; and induced oxidative stress. ATPase activities and the expressions of energy metabolism-related genes were significantly decreased. Also, the expressions of related necroptosis (RIPK1, RIPK3, MLKL, TAK1, TAB2, and TAB3) were significantly increased, and the MAPK pathway was activated. Besides, H_2_S exposure activated the NF-*κ*B classical pathway and induced TNF-*α* and IL-1*β* release. Taken together, we conclude that H_2_S exposure induces oxidative stress and energy metabolism dysfunction; evokes necroptosis; activates the MAPK pathway, eventually triggering the NF-*κ*B pathway; and promotes inflammatory response in chicken spleens.

## 1. Introduction

Like PM2.5, carbon monoxide (CO), sulfur dioxide (SO_2_), and other atmospheric pollutants, H_2_S is one of the main pollutants in the atmosphere [1, 2]. H_2_S in the environment mainly comes from crude oil, natural gas, volcanic gas, and wetlands, and it is often produced in various occupational environments, such as leather tanning, rubber vulcanization, and synthetic fiber and paper making [3]. The adverse effects of H_2_S on humans have been affirmed, including its respiratory toxicity, neurotoxicity, and immunotoxicity [4, 5]. Some workers in this occupational environment are inevitably exposed to H_2_S, which seriously affects human health. Among workers with long exposure to H_2_S, the expression level of proinflammatory interleukin- (IL-) 8 was increased significantly [6], suggesting the immune injury of H_2_S exposure. Sreejai and Jaya reported that H_2_S exposure induced oxidative stress and weakened antioxidant ability in fishes [7]. In addition, H_2_S is one of the harmful gases that the poultry industry pays close attention to; it has been reported that H_2_S in chicken houses significantly reduced meat quality and the laying rate of broilers [8]. The damaging effects of excessive H_2_S exposure on the trachea and jejunum of chickens have also been suggested [9, 10].

Necroptosis is a newly discovered pathway of regulated necrosis that is mediated by the proteins of receptor interacting protein kinase-1 (RIPK1), RIPK3, and mixed lineage kinase domain-like (MLKL), and caspase-8 has been confirmed as the most crucial factor for preventing necroptosis by cleaving RIPK1 and RIPK3 [11]. Recent studies have provided that necroptosis is regulated by various mechanisms. Reactive oxygen species (ROS) could involve in high glucose-induced necroptosis [12]. Hemin-induced necroptosis was accompanied by the rapid depletion of intracellular glutathione (GSH) [13]. Similarly, Yang et al. suggested that selenium deficiency-induced RIPK3-dependent necroptosis in cardiomyocytes was accompanied with oxidative stress and activated the mitogen-activated protein kinase (MAPK) pathway [14]. Interestingly, there is novel evidence which showed that RIPK3 could activate pyruvate dehydrogenase complex (PDHX) in tumor necrosis factor- (TNF-) induced necroptosis, and upon activation, PDHX enhanced mitochondrial ROS production [15]. Also, the change of ATPase activities was found during necroptosis [16]. This suggests that necroptosis may be associated with energy metabolism. A previous study has shown that TNF signaling is a key regulator involved in necroptosis [17]. Also, the release of proinflammatory mediators was observed in environmental challenge-induced necroptosis [18, 19]. Furthermore, the regulation of necroptosis in immune tissues and cells was also suggested. Particles and cigarette smoke extract could induce neutrophil necroptosis through the RIPK1/RIPL3/MLKL signaling pathway [20, 21]. Bacterial-induced necroptosis was targeted to splenic macrophages, and the loss of macrophages affected the host's ability to regulate the inflammation [22]. On the other hand, accumulating evidence has demonstrated that oxidative stress and inflammation have been considered a driving force for necroptosis [23]. We all know that heat shock proteins (HSPs) could alleviate inflammatory-induced injury through inhibiting ROS and cytokines. Zhao et al. found that in TNF-induced necroptosis, HSP90 was required for modulating the stability of MLKL, which plays a crucial role in necroptosis execution [24]. Besides, some people think that HSP70 could reduce the injury of the tissue and cell by inhibiting the activity of nuclear factor-*κ*B (NF-*κ*B), one of the important upstream signals of cytokine expression [25], and HSP70 also plays a previously unrecognized and important role in suppressing RIPK1-dependent necroptosis [26].

As a noxious, toxic gas produced by organic decomposition and by many industrial processes, H_2_S exposure could seriously affect the immune function of organisms [27, 28]. The spleen is one of the peripheral immune organs and the largest lymphoid organ of an organism; it plays an important role in maintaining the immune function. In order to investigate the possible mechanisms of excessive H_2_S exposure-induced necroptosis, light and transmission electron microscopy, qRT-PCR, western, and kits were performed to detect the levels of related necroptosis, ATPases and antioxidative enzymes, related energy metabolism, and cytokines (TNF-*α* and IL-1*β*). Our results would reveal the roles of the MAPK pathway on H_2_S-induced necroptosis in the chicken spleens. Furthermore, the associated health risks are essential for effective environmental management and mitigation policies.

## 2. Materials and Methods

### 2.1. Preparation of Animals

Seventy-two 1-day-old Ross 308 male broilers (Weiwei Co. Ltd., Harbin, China) were housed in two environmentally controlled rooms and were divided into the following two groups. The control group broilers were raised in a separate chamber without H_2_S. The H_2_S group broilers were exposed to 4.0 ± 0.5 ppm, at 0-3 weeks of age, and 20.0 ± 0.5 ppm, at 4-6 weeks of age. The conditions of both chambers and the compositions of the diets (shown in Table S1) have been previously described [29]. At 2, 4, and 6 weeks, the broilers were euthanized by cervical dislocation and the spleen tissues were quickly collected (*n* = 10 per group, the remaining two chickens in each group were on standby for any unexpected condition). The tissues were excised immediately on ice, washed in a physiological saline solution (PBS), and then stored at -80°C.

### 2.2. Histopathological and Ultrastructural Examinations

The histopathological and ultrastructural changes of spleen tissues were performed as described [30]. The histopathological and ultrastructural changes were observed under a light microscopy and a transmission electron microscope (Hitachi 7650, Tokyo, Japan), respectively.

### 2.3. RNA Isolation and qRT-PCR Analysis

Total RNA was isolated from the tissues using TRIzol reagent, and reverse cDNA was carried out with the First-Strand cDNA Synthesis Kit (TIANGEN Biotech Co. Ltd., Beijing). The primers for the detection of target mRNA are shown in Table 1. Gene expression levels were evaluated by qRT-PCR as previously reported [29]. The mRNA relative levels were calculated according to the 2^-*ΔΔ*Ct^ method. *β*-Actin served as the endogenous controls for normalization.

### 2.4. Western Blot Analysis

The western blot analysis of proteins were performed as previously reported [29]. The antibodies (NOX2, JNK, ERK, and p38) were purchased from Beijing Bioss Biotechnology Co. Ltd. The dilution ratio of primary antibodies is shown in Table S2. The GAPDH content was analyzed as the loading control with rabbit polyclonal antibody (Sigma-Aldrich, USA).

### 2.5. Determination of Oxidative Stress

The oxidative stress markers (GSH, CAT, T-AOC, SOD, iNOS, NO, H_2_O_2_, and MDA) of spleen tissues were detected by the appropriate assay kits (Nanjing Jiancheng Bioengineering Institute, China), according to the method of Hu et al. [30].

### 2.6. Determination of ATPase Activities

The activities of Na^+^-K^+^-ATPase, Ca^2+^- Mg^2+^-ATPase, Ca^2+^-ATPase, and Mg^2+^-ATPase were determined using the appropriate assay kits (Nanjing Jiancheng Bioengineering Institute, China) and were measured as previously described [31].

### 2.7. Statistical Analysis

Statistical analyses of all data were conducted using GraphPad Prism software (version 7.0, GraphPad Software Inc., San Diego, CA, USA). The software showed a normal distribution and passed equal variance testing. The differences between the means of the C group and the H_2_S group were analyzed by 2-way ANOVA with Tukey test. The bars represent the means ± SD of 10 individuals (*n* = 10). *p* < 0.05 was considered a statistically significant difference.

## 3. Results

### 3.1. Histopathological and Ultrastructural Changes in Chicken Spleens

**Figure 1:**
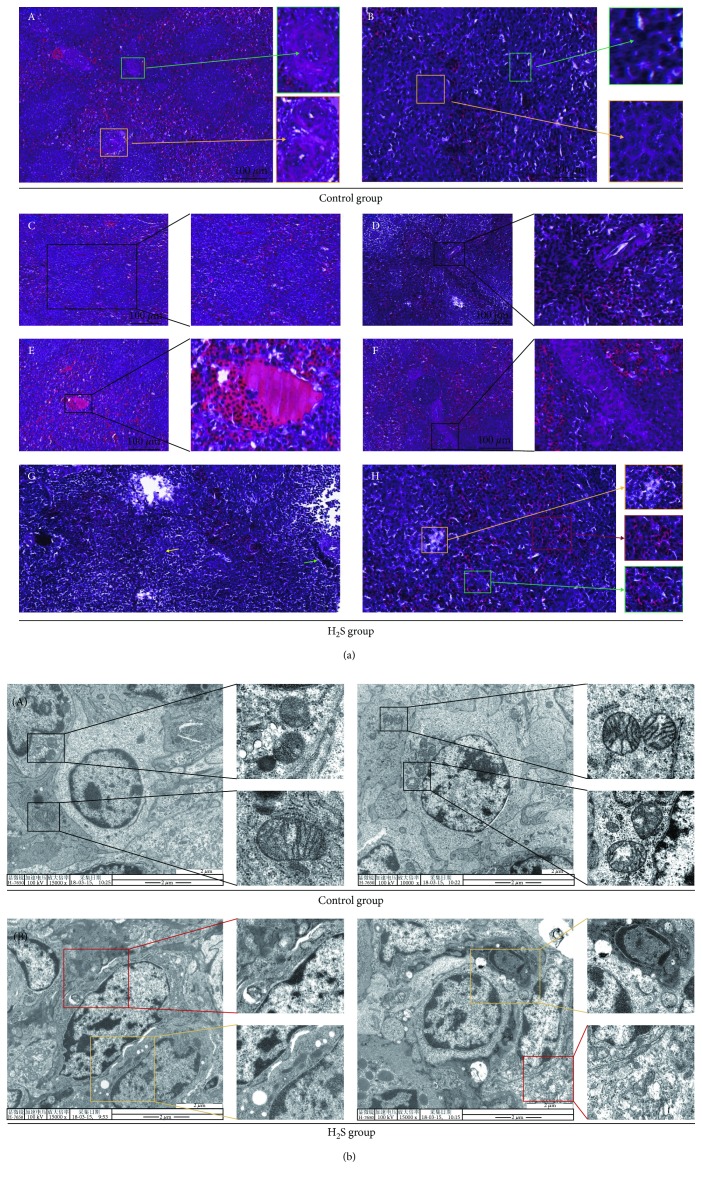
Histopathological and ultrastructural changes in chicken spleens. Histopathological changes and ultrastructural changes in chicken spleen tissues after 6 weeks of H_2_S exposure. (a) represents histopathological changes of chicken spleen tissues (400x). A: the normal spleen cells including the central artery (yellow box) and trabecula (green box). B: abundant immune cells in the control spleen including lymphocytes (green box) and macrophages (yellow box). C–F: the spleen damage features: white pulp hyperplasia (C), multiplied lymphatic nodules (D), red pulp congestion (E), splenic cord hyperplasia (F). G: reduced lymphocytes (green arrow) and macrophages (yellow arrow). H: spleen cell necrotic features: karyorrhexis (green box), karyolysis (yellow box), and hematocytosis (red box). (b) represents ultrastructural changes of chicken spleen tissues. A: the normal mitochondrial structure (black box). B: spleen cell necrotic features: mitochondria swelling and vacuolation (yellow box), cell membrane breakage, dissolution, and cytosolic content spillover, accompanied with extensive formation of vesicles (red box).

We observed spleen tissues stained by H&E in the control groups and H_2_S groups at 6 weeks (Figure 1(a)). The spleen tissues in the control group displayed normal morphologies, including the following: the boundary of the red pulp and the white pulp is clear, the central artery is obvious (Figure 1(a), A, yellow box), the trabecula is clear (Figure 1(a), A, green box), the white pulp lymphocytes are abundant (Figure 1(a), B, green box), and the macrophages are abundant (Figure 1(a), B, yellow box). However, many typical spleen damage features appeared in the tissues of the H_2_S group: spleens suffering from atmospheric H_2_S exposure showed white pulp hyperplasia (Figure 1(a), C), red pulp congestion was observed (Figure 1(a), E), the red pulp area had splenic cord hyperplasia (Figure 1(a), F), and lymphatic nodules multiplied (Figure 1(a), D). The number of lymphocytes (Figure 1(a), G, green arrow) and macrophages (Figure 1(a), G, yellow arrow) in the H_2_S group spleen decreased significantly compared with the control group spleen. In addition, some necrotic features were also observed in the H_2_S group spleen, including karyorrhexis (Figure 1(a), H, green box), karyolysis (Figure 1(a), H, yellow box), and hematocytosis (Figure 1(a), H, red box).

In addition, we also observed the ultrastructural changes in spleen cells. As shown in Figure 1(b), there were no obviously visible ultrastructural changes in the control group wherein the cell membrane was holonomic, the mitochondria were rich, and cristae were complete (Figure 1(b), A). Contrastingly, the H_2_S-exposed spleen cells showed typical necrosis characteristics, like mitochondria swelling and even vacuolation (Figure 1(b), B, yellow box), cell membrane breakage, dissolution, and cytosolic content spillover, accompanied with extensive formation of vesicles (Figure 1(b), B, red box). All these observations confirmed that atmospheric H_2_S exposure induces necrosis accompanied with inflammation in the spleens.

### 3.2. The Relative Expressions of Related Necroptosis in Chicken Spleens

**Figure 2:**
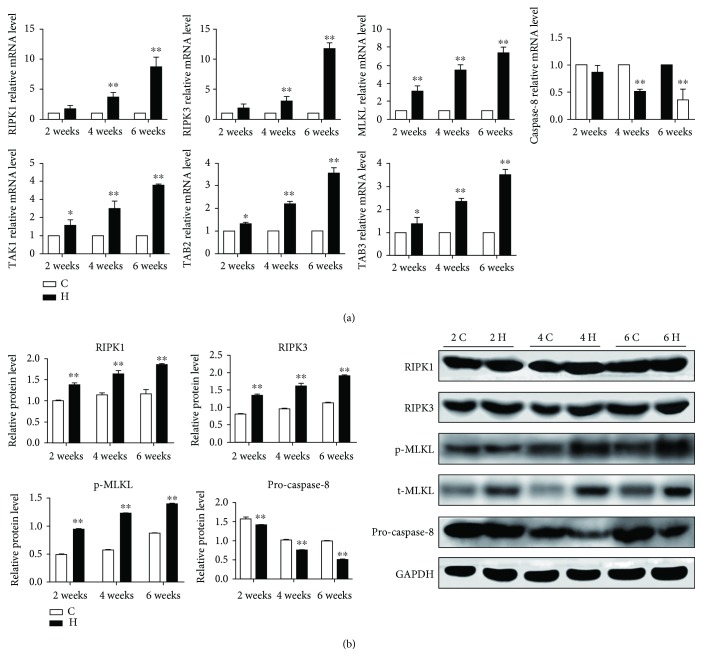
The relative expressions of related necroptosis in chicken spleens. The effect of H_2_S on the mRNA (a) and protein (b) expressions of related necroptosis in chicken spleens. The results are from at least three independent experiments. Data are represented as the means ± SD (*n* = 10). *β*-Actin and GAPDH were selected as the reference of mRNA and protein expressions, respectively. ∗ shows significant difference from the corresponding control groups (*p* < 0.05); ∗∗ shows significant difference from the corresponding control groups (*p* < 0.01).

Atmospheric H_2_S is well known as an important factor inducing necrosis. We next investigated the type of necrosis caused by H_2_S exposure; we determined necroptosis-related genes through running qRT-PCT and western blot analysis. As shown in Figure 2(a), the mRNA expressions of RIPK1, RIPK3, MLKL, TGF-beta-activated kinase 1 (TAK1), abdominal B2 (TAB2), and TAB3 were significantly increased while the caspase-8 mRNA level was significantly decreased (*p* < 0.05 or *p* < 0.01) in H_2_S groups compared with the corresponding control groups at 2, 4, and 6 weeks. However, there was no significance of RIPK1, RIPK3, and caspase-8 between the control group and the H_2_S group at 2 weeks. In addition, H_2_S exposure increased the protein expressions of RIPK1, RIPK3, and the phosphorylated MLKL, while pro-caspase-8 was inhibited (*p* < 0.05 or *p* < 0.01), reflecting the occurrence of necroptosis induced by H_2_S exposure (Figure 2(b)).

### 3.3. The Antioxidant Capacity in Chicken Spleens

**Figure 3:**
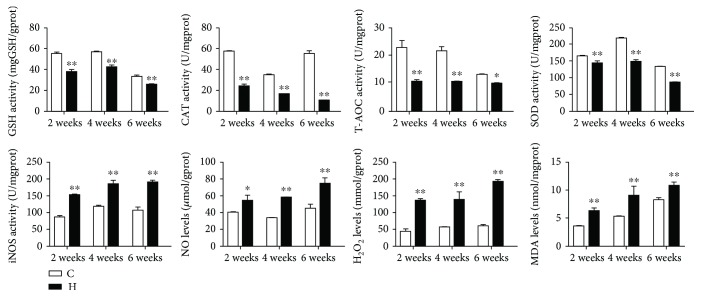
The antioxidant capacity in chicken spleens. The effect of H_2_S on the antioxidant capacity in chicken spleens. They represent GSH, CAT, T-AOC, SOD and iNOS activities, and NO, H_2_O_2_ and MDA contents. ∗ shows significant difference from the corresponding control groups (*p* < 0.05); ∗∗ shows significant difference from the corresponding control groups (*p* < 0.01).

To determine the effect of H_2_S gas on the antioxidant capacity in chicken spleens, we measured the activities of GSH, CAT, T-AOC, SOD, and iNOS and the contents of NO, H_2_O_2_, and MDA (Figure 3). Compared with the corresponding control groups, the activities of GSH, CAT, T-AOC, and SOD were significantly decreased (*p* < 0.05 or *p* < 0.01) in the H_2_S groups at all time points. However, the iNOS activity and the contents of NO, H_2_O_2_, and MDA were significantly upregulated (*p* < 0.05 or *p* < 0.01) in the H_2_S-exposed chicken spleens. In addition, H_2_S exposure-induced variation tendencies of oxidation resistance indexes were in a time-dependent manner.

### 3.4. The ATPase Activities and Energy Metabolism-Related Expressions in Chicken Spleens

**Figure 4:**
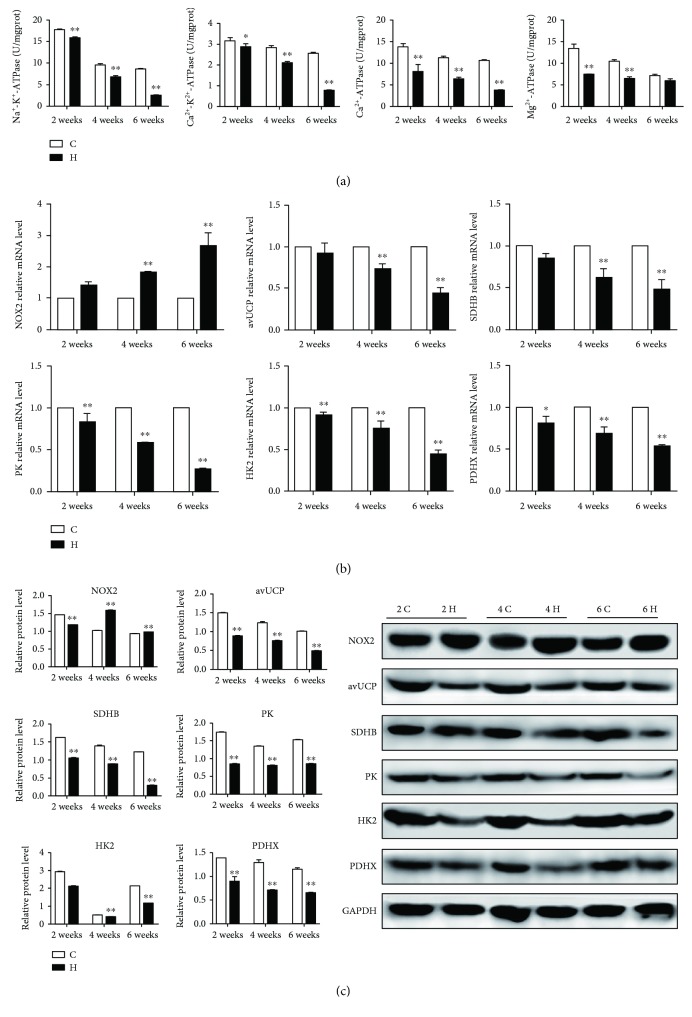
The ATPase activities and energy metabolism-related expressions in chicken spleens. The effect of H_2_S on energy metabolism of chicken spleens. (a) represents the activities of Na^+^-K^+^-ATPase, Ca^2+^-Mg^2+^-ATPase, Ca^2+^-ATPase, and Mg^2+^-ATPase of spleen tissues. (b) and (c) represent the mRNA and protein expressions of spleen tissues, respectively. The results are from at least three independent experiments. Data are represented as the means ± SD (*n* = 10). *β*-Actin and GAPDH were selected as the reference of mRNA and protein expressions, respectively. ∗ shows significant difference from the corresponding control groups (*p* < 0.05); ∗∗ shows significant difference from the corresponding control groups (*p* < 0.01).

Mitochondria are an important energy metabolic site; their damage means that energy metabolism may be impaired. To understand whether H_2_S gas could affect energy metabolism of chicken spleens, we detected the activities of Na^+^-K^+^-ATPase, Ca^2+^-Mg^2+^-ATPase, Ca^2+^-ATPase, and Mg^2+^-ATPase as shown in Figure 4(a); the reduction of ATPase activities was found in H_2_S-exposed spleens compared with the corresponding control groups (*p* < 0.05 or *p* < 0.01). Notably, the downward trend of Na^+^-K^+^-ATPase activity at 2, 4, and 6 weeks was the most obvious. However, there was no significance of the Mg^2+^-ATPase activity at 6 weeks between the control group and the H_2_S group (*p* > 0.05). In addition, we also carried out qRT-PCR and western blot to verify the expressions of NOX2, avUCP, SDHB, PK, HK2, and PDHX to confirm energy metabolism dysfunction. As shown in Figures 4(b) and 4(c), the expressions of avUCP, SDHB, PK, HK2, and PDHX were markedly decreased, while NOX2, as a key regulator of oxygen free radicals, was elevated significantly under H_2_S exposure (*p* < 0.05 or *p* < 0.01). Based on our results, we conclude that H_2_S could induce energy metabolism dysfunction in chicken spleens.

### 3.5. The Activation of the MAPK Pathway

**Figure 5:**
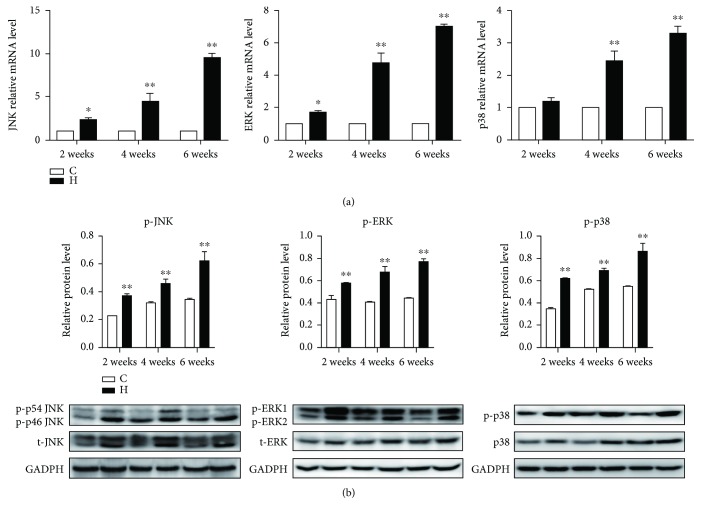
The activation of the MAPK pathway. The effect of H_2_S on MAPK activation in chicken spleens. (a) and (b) represent the effect of H_2_S on the mRNA (a) and phosphorylated protein (b) expressions of JNK, ERK, and p38 in chicken spleens. The results are from at least three independent experiments. Data are represented as the means ± SD (*n* = 10). *β*-Actin and total JNK, total ERK, and total p38 were selected as the reference of mRNA and phosphorylated protein expressions, respectively. ∗ shows significant difference from the corresponding control groups (*p* < 0.05); ∗∗ shows significant difference from the corresponding control groups (*p* < 0.01).

The MAPK pathway is an important signal pathway which could regulate various pathological mechanisms, including oxidative stress, energy metabolism dysfunction, and inflammation. To assess the relationship between necroptosis and the MAPK pathway, we measured the expressions of JNK, ERK, and p38 in chicken spleens exposed to H_2_S gas. As is presented in Figure 5, the expressions of both the mRNA and phosphorylated proteins of JNK, ERK, and p38 were elevated (*p* < 0.05 or *p* < 0.01) in H_2_S-exposed chicken spleens, and with the time prolongation of H_2_S exposure, the expression of the MAPK pathway became higher and higher. This suggests that H_2_S exposure could activate the MAPK pathway.

### 3.6. The Activation of the NF-*κ*B Pathway and the Level of Inflammatory Response in Chicken Spleens

**Figure 6:**
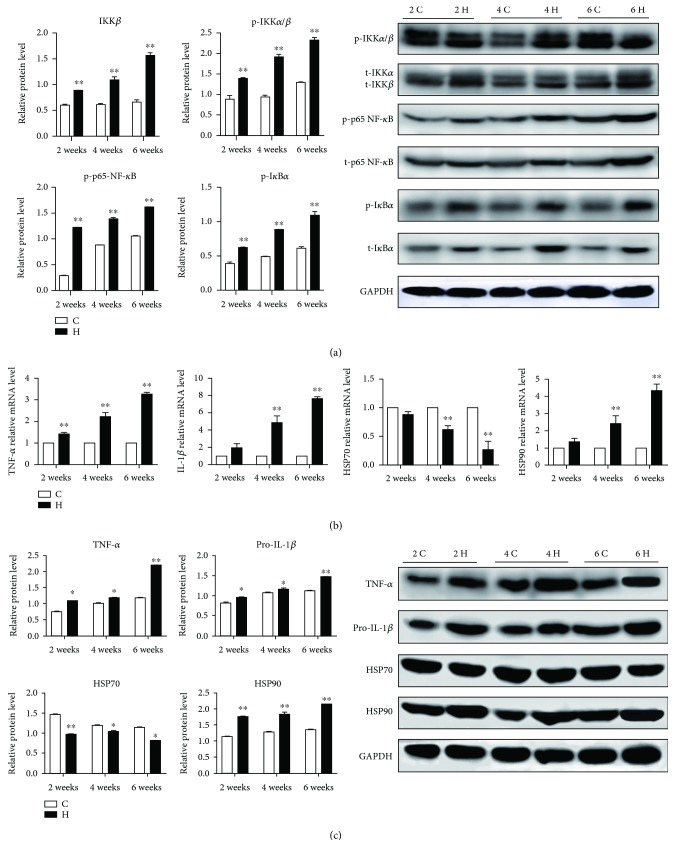
The activation of the NF-*κ*B pathway and the expressions of inflammatory factors and HSP in chicken spleens. The effect of H_2_S on the expressions of the NF-*κ*B pathway (a) and mRNA (b) and protein (c) expressions of TNF-*α*, IL-1*β*, HSP70, and HSP90 in chicken spleens. The results are from at least three independent experiments. Data are represented as the means ± SD (*n* = 10). *β*-Actin and GAPDH were selected as the reference of mRNA and protein expressions, respectively. ∗ shows significant difference from the corresponding control groups (*p* < 0.05); ∗∗ shows significant difference from the corresponding control groups (*p* < 0.01).

Many researches have suggested that necroptosis has a regulation on inflammation. Lys63 polymerization of RIPK1 could promote NF-*κ*B pathway activation. Phosphorylation of serine 536 at NF-*κ*B p65 is an important marker of activation of the NF-*κ*B signaling pathway. As shown in Figure 6(a), the expressions of IKK*α*/*β* and p65 NF-*κ*B in the chicken spleen tissues were significantly increased after 2, 4, and 6 weeks of H_2_S exposure (*p* < 0.05 or *p* < 0.01). In addition, I*κ*B*α* was degraded (*p* < 0.05) in the H_2_S group spleens. However, H_2_S exposure had no significant effect on IKK*α* in the NF-*κ*B nonclassical pathway (*p* > 0.05).

The NF-*κ*B signaling pathway is an important pathway for the regulation of inflammatory response [32]. Therefore, we further examined the downstream gene expressions of the NF-*κ*B pathway. As shown in Figures 6(b) and 6(c), the expressions of proinflammatory cytokines TNF-*α* and IL-1*β* and highly conserved protein HSP90 were upregulated obviously while HSP70 was downregulated in H_2_S-exposed chicken spleens (*p* < 0.05 or *p* < 0.01). Furthermore, the trend of the mRNA expressions of the NF-*κ*B pathway, TNF-*α*, IL-1*β*, HSP70, and HSP90 was presented in a H_2_S-exposed time-dependent manner. Taken together, inflammatory response could involve in atmospheric H_2_S-induced necroptosis in chicken spleens.

## 4. Discussion

H_2_S is one of the main air pollutants, and its exposure can cause extensive toxicity to both humans and animals. Current studies about the pathological mechanisms of excessive H_2_S-induced immune injury are mainly concerned on apoptosis and autophagy. However, necroptosis is a regulated form of necrosis, which can trigger innate immune responses. Thus, as an important mechanism which could mediate immune function, it is essential to assess the possible molecular mechanisms of necroptosis caused by H_2_S exposure, and that is also what this experiment is about.

Much of our knowledge of necroptosis is that it is mediated by RIPK1, RIPK3, and MLKL during caspase-8 inhibition. There are reports suggesting that environmental challenges could induce the occurrence of necroptosis. Rainbow trout cells exposed to cadmium showed lost plasma membrane integrity and displayed cell swelling, signs associated with secondary necrosis, or, equally possible, necroptotic cell death [33]. A typical environmental carcinogen, benzo(a)pyrene, also induced necroptotic cell death via the mitochondrial pathway in the lung carcinoma cell lines [34]. Furthermore, aluminum exposure increased the RIPK1 level, and the administration of necrostatin-1 (Nec-1) decreased the neural cell death [35]. The lung epithelial cell which suffers from cigarette smoke exposure exhibited mitochondrial damage, and the expressions of RIPK1, RIPK3, and MLKL were elevated [36]. Yang et al. inferred that Se deficiency induced chicken cardiomyocyte necrosis and increased the levels of TAB and TAK1 [14]. In the present study, we observed the characteristics of cell necrosis such as nuclear dissolution and organelle swelling. Further detection at the molecular level show that the expressions of related necroptosis were upregulated significantly while caspase-8 was downregulated in chicken spleens, suggesting that excessive H_2_S exposure could induce necroptosis of chicken spleens, and the degree of this damage is time-dependent exposure.

Environmental challenge exposure, such as cadmium, arsenic, and lead, could induce oxidative stress through elevating ROS and MDA levels, decreasing the activities of glutathione peroxidase (GPX) and SOD [37–39]. Besides, environmental gases, such as PM2.5, cigarette smoke, NH_3_, SO_2_, nitrogen dioxide (NO_2_), and ozone (O_3_), could lead to oxidative stress, and the mitochondria were also damaged by varying degrees, in more detail, of changes manifested as the increased levels of MDA and the decreased levels of total superoxide dismutase (T-SOD) [40–45]. In addition, Han et al. have illustrated that oxidative stress could induce necroptosis during hyperoxic acute lung damage [46]. Besides, the activation of the NADPH oxidase systems or inhibition of mitochondrial respiration lead to the formation of a lot of H_2_O_2_ in long-term H_2_S-exposed human red blood cells [47]. Here we have shown that, in contrast to control groups, the antioxidant ability was significantly impaired in H_2_S groups, and with the prolongation of the H_2_S exposure time, the antioxidation ability became weaker and weaker. From this perspective, our findings supported that excessive H_2_S exposure could induce oxidative stress, and this toxicological mechanism is involved in H_2_S-induced necroptosis.

The preceding ultrastructural observation has suggested that the mitochondria were damaged, such as mitochondrial cristae break, mitochondria swelling, and vacuolation. On the other hand, mitochondria are the main energy production site, and their damage means energy metabolism may be dysfunctional. Indeed, in the current study, compared with the corresponding control groups, the levels of energy metabolism-related genes were downregulated with atmospheric H_2_S exposure. Of more interest, NOX2, as an important enzyme which could promote the production of ROS and oxygen free radicals, was upregulated significantly following H_2_S exposure. In addition, we also demonstrated that excessive H_2_S exposure suppressed the ATPase activities, and with the increase of the H_2_S exposure time, the activities of ATPase were becoming lower and lower. The finding showed that neoalbaconol-induced necroptosis resulted in energy depletion by downregulated HK1 and HK2 levels [48]. Similarly, pyruvate has a protective role in ischaemic enterocytes; the level of pyruvate was depressed during necroptosis [49]. Moreover, the impairment of Na^+^/K^+^-ATPase was detected in cell necrosis [50], and the ATPase viabilities were inhibited obviously during necroptosis [16]. These fit well with the implications of our findings and suggest that excessive atmospheric H_2_S exposure may lead to energy metabolism dysfunction in chicken spleens in a time-dependent manner.

It is also relevant to note that the MAPK pathway is an important signaling pathway that regulates various pathological mechanisms that is comprised of ERK, p38, and JNK. Viewed in this light, our study focused on whether atmospheric H_2_S could active the MAPK pathway and then mediate necroptosis. Results indicated that the levels of ERK, p38, and JNK were all elevated under H_2_S exposure, suggesting that the MAPK pathway was activated and that regulated necroptosis was induced by atmospheric H_2_S exposure. Support for our observations come from a study by Xie et al. who reported that an increase of ROS production and a depletion of GSH induced by dimethyl fumarate were found and MAPK activation is involved in DMF-induced necroptosis [51]. The activation of TAK1 and I*κ*B kinase (IKK) complex signal also activated the kinases JNK, p38, and ERK as well as NF-*κ*B transcription factors, culminating in the expression of proinflammatory genes [52].

The important functions of necroptosis in inflammation have been reported, and it has been suggested that it could be implicated in the pathogenesis of many inflammatory diseases, and cytokines are important indicators that reflect inflammation [53]. We have found the pathological changes of inflammation in chicken spleens, such as inflammatory infiltration, coagulation necrosis, and liquefaction necrosis under excessive H_2_S exposure. In addition, compared with control groups at various time points, the higher levels of proinflammatory cytokines TNF-*α*, IL-1*β*, and NF-*κ*B in H_2_S groups were observed. These all provided a sign that the occurrence of necroptosis was accompanied with inflammation. The study supporting the immune injury of H_2_S and lead exposure come from Wang et al. and Li et al., which showed the release of cytokines including IL-4, IL-6, TNF-*α*, and IL-1*β* through activation of the NF-*κ*B pathway [54, 55]. Besides, several studies have shown that RIPK3-medicated necroptosis promotes inflammation through increasing cytokine and inflammasome molecule levels [56, 57]. Furthermore, we all know that HSPs as a class of highly conserved proteins could involve in inflammatory response, and it has been reported that HSPs could also regulate oxidative stress and immune imbalance in copper- and arsenic-exposed chicken intestines [58]. Chen et al. have demonstrated that HSP70 inhibited NF-*κ*B in a cadmium-exposed chicken spleen [25], and a recent study has revealed that HSP70 could suppress RIPK1-dependent necroptosis [26]. In addition, the level of HSP90 was increased in TNF-induced necroptosis, because HSP90 could make MLKL more stable [24], which is consistent with our results. However, Zhang et al. found that the HSP90 inhibitor DHQ3 upregulated the expression of RIPK1, RIPK3, and MLKL, while caspase-8 was nearly undetected in cells [16]. The regulation of HSP90 on necroptosis needs to be further investigated.

In conclusion, here we have provided evidence to argue that excessive atmospheric H_2_S exposure induced oxidative stress and energy metabolism dysfunction, evoked necroptosis and activated the MAPK pathway, trigged the NF-*κ*B pathway, and promoted inflammatory response in chicken spleens, and the pathological process induced by H_2_S is in a time-dependent manner. Accordingly, these results will provide valuable clues for immune damage induced by atmospheric H_2_S in addition to the respiratory system. Evaluating and monitoring the concentrations of atmospheric H_2_S in chicken houses and other breeding houses and the associated health risks are essential for effective environmental management and mitigation policies.

## Figures and Tables

**Table 1:** mRNAs primer sequences.

Gene	Forward 5′ to 3′	Reverse 5′ to 3′
RIPK1	AAGGGCGTTTCATCCTGGAG	CGGCAGGTCTCTTCTTTGGT
RIPK3	CCCATGGACAGGGAATGGAA	CCACAAGTCTCTGGTAGCGG
MLKL	CCATGGGTGGTTCCTCCTTC	TGGATCTTCCGCACCTTAGC
Caspase-8	CCGATTCTCTGGGCAACTGT	ATCCACATGTGTCCCGTTCC
JNK	TGACCGAGTGAGGAGACGAT	ACTGTATCGAACGCAGCACA
ERK	AGAATCTCACAGCGTCTCGC	GGTGTGATTCATCAGCATCTTCA
p38	GCGAGTCCCTAATGCCTACG	ACAACTGTTGAGCCACACTCA
TAK1	ACCGGGTTAAACGGATCCAC	TCGTTTTGCTCGTGCTTTGG
TAB2	CTCTTTTTCCTTGGCGAGCG	GCTTCCTTGGGCCATTCGTA
TAB3	TTGAACCACCGCAAAGACCT	GGTTTGGGTTGACCCGACAT
TNF-*α*	GCCCTTCCTGTAACCAGATG	ACACGACAGCCAAGTCAACG
IL-1*β*	ACTGGGCATCAAGGGCTACA	GCTGTCCAGGCGGTAGAAGA
NF-*κ*B	TCAACGCAGGACCTAAAGACAT	GCAGATAGCCAAGTTCAGGATG
HSP70	CGGGCAAGTTTGACCTAA	TTGGCTCCCACCCTATCTCT
HSP90	TCCTGTCCTGGCTTTAGTTT	AGGTGGCATCTCCTCGGT
NOX2	GGACTGTCCATCTTTGTCGT	TTACACGGGTAGAGCAGCAC
PK	AGCAGCAGGAGACACCGAAC	TGAGGCGGGCAACATTCAT
HK2	TTCGACCACATCGTCCACTG	ACCACGTCCAGGTCAAACTC
SDHB	CGGTCCAGGGGATCTGTCG	AGATGCCTTCCCTGCATGAC
PDHX	ACGCTTGGGCTCCCTAATTG	TCCACTTTAGGAGGGGCAGA
avUCP	GATGCAGAGAAACAGAGCGG	AAGGTGCAGAGGTCAGCGAT
*β*-Actin	CCGCTCTATGAAGGCTACGC	CTCTCGGCTGTGGTGGTGAA

## Data Availability

The data used to support the findings of this study are available from the corresponding authors upon request.

## Supplementary Materials

Supplementary Materials Table S1 shows the ingredients and chemical composition of the basal diets, and we refer to this table in Section 2.1 of the manuscript. Table S2 is the dilution ratio of primary antibodies, and we refer to this table in Section 2.4 of the manuscript.
