# Supplementary material for: Hydrogen Sulfide Gas Exposure Induces Necroptosis and Promotes Inflammation through the MAPK/NF-κB Pathway in Broiler Spleen
Source: Oxid Med Cell Longev. 2019 Jul 31;2019:8061823. doi: 10.1155/2019/8061823 (PMC6701317; doi:10.1155/2019/8061823)
Supplement: Supplementary Materials — Table S1 shows the ingredients and chemical composition of the basal diets, and we refer to this table in Section 2.1 of the manuscript. Table S2 is the dilution ratio of primary antibodies, and we refer to this table in Section 2.4 of the manuscript. [file 8061823.f1.doc]

**Table S1.** **Ingredients and chemical composition of the basal diets**

| Items  Ingredient (%, as-fed basis) | Diet 1 (0 − 14 d)  (21% protein) | Diet 2 (15 − 28 d)  (19% protein) |
| --- | --- | --- |
| Corn | 45.69 | 51.05 |
| Wheat | 15.60 | 15.64 |
| Soybean meal, deshelled | 24.60 | 17.31 |
| Rapeseed meal | 2.30 | 3.20 |
| Corn gluten | 2.50 | 3.20 |
| Fish meal | 4.60 | 3.30 |
| Meat and bone meal |  | 1.00 |
| Limestone | 0.93 | 0.80 |
| TCP | 0.55 | 0.45 |
| Salt | 0.25 | 0.25 |
| Animal fat | 1.95 | 2.70 |
| L-Lysine·HCl | 0.03 | 0.05 |
| DL-Methionine |  | 0.05 |
| Vitamin-mineral premix^1^ | 1.00 | 1.00 |
| Calculated analysis ME, kcal/kg | 2,959.50 | 3,102.20 |
| Protein | 21.76 | 19.03 |

Supplied the following per kilogram of diet: vitamin A, 12,500 IU; vitamin D, 2,500 IU; vitamin E, 18.75 mg; vitamin K3, 2.65 mg; vitamin B1, 2 mg; vitamin B2, 6 mg; vitamin B12, 0.025 mg; D-biotin, 0.325 mg; folic acid, 1.25 mg; d-pantothenic acid, 12 mg; nicotinic acid, 50 mg.

**Table S2. The dilution ratio of primary antibodies**

| **Genes name** | **Dilution ratio** |
| --- | --- |
| RIPK1 | 1:500 |
| RIPK3 | 1:500 |
| MLKL | 1:500 |
| p-MLKL | 1:500 |
| pro-Caspase-8 | 1:500 |
| NOX2 | 1:400 |
| avUCP | 1:2000 |
| SDHB | 1:500 |
| PK | 1:1500 |
| HK2 | 1:1000 |
| PDHX | 1:500 |
| IKKα/β | 1:500 |
| p-IKKα/β | 1:500 |
| p65 NF-κB | 1:400 |
| p-p65 NF-κB | 1:500 |
| IκBα | 1:500 |
| p-IκBα | 1:500 |
| TNF-α | 1:500 |
| pro-IL-1β | 1:500 |
| HSP70 | 1:500 |
| HSP90 | 1:500 |
| JNK | 1:500 |
| p-JNK | 1:500 |
| ERK | 1:500 |
| p-ERK | 1:500 |
| p38 | 1:500 |
| p-p38 | 1:500 |
| GAPDH | 1:500 |
